# The heterogeneity of negative problem orientation in Chinese adolescents: A latent profile analysis

**DOI:** 10.3389/fpsyg.2022.1012455

**Published:** 2022-10-05

**Authors:** Rong-Mao Lin, Xia-Xin Xiong, Yi-Lin Shen, Nan Lin, Yan-Ping Chen

**Affiliations:** ^1^School of Psychology, Fujian Normal University, Fuzhou, Fujian, China; ^2^Department of Social Psychology, University of Groningen, Groningen, Netherlands

**Keywords:** negative problem orientation, adolescents, latent profile analysis, cognitive vulnerabilities, psychological symptoms

## Abstract

Negative problem orientation (NPO) has become an essential construct for comprehending social problem-solving deficits. However, the heterogeneity of NPO has not yet been explored. With a sample of Chinese adolescents (*N* = 2,174), four latent profiles were identified as *lower NPO*, *moderate NPO*, self-inefficacy and negative outcome expectancy (*SI&NOE*), and *dysfunctional NPO*. Compared to the *lower NPO* and *moderate NPO*, a greater percentage of boys in the *SI&NOE* and *dysfunctional NPO* profiles than were girls. In addition, lower grades and younger adolescents tended to engage in the *moderate NPO* and *SI&NOE* profiles. The *dysfunctional NPO* reported higher levels of worry, depressive symptoms, anxiety, and stress, and worse sleep quality than the other profiles. The implications of these findings are discussed herein.

## Introduction

Negative problem orientation (NPO) has increasingly become an essential construct for understanding deficits in social problem-solving ability. It is known for causing negative emotions and avoidance motivation that inhibits subsequent problem-solving endeavors (Lee and Woodruff-Borden, 2018; Söğüt et al., 2021). According to social problem-solving theory, NPO refers to a comprehensively disruptive cognitive-emotional set and/or attitude toward social problems encountered, increasing useless worry about those problems and inhibiting the establishment of effective resolutions (Nezu, 2004). Specifically, it involves three components: (a) view of a problem as a significant threat to wellbeing (including psychological, social, and economic), (b) doubt about one’s own personal ability to solve problems successfully, and (c) easily becoming frustrated and upset when confronted with social problems (D’Zurilla et al., 2004; Nezu et al., 2013).

The vital role NPO plays in adolescent psychotherapy and mental health is well-recognized. For example, previous studies have shown that NPO increases adolescent worry and anxiety (Donovan et al., 2016; Kasik et al., 2017; Lee and Woodruff-Borden, 2018), reduces the effort expended to solve social problems (Chu et al., 2017), and thus leads to several psychopathological issues such as generalized anxiety disorder (GAD) (Koerner et al., 2015), social anxiety disorder (SAD) (Hearn et al., 2017), obsessive–compulsive disorder (OCD) (Fergus and Wu, 2010), and major depressive disorder (MDD) (Becker-Weidman et al., 2010). These results were mainly obtained from a variable-oriented perspective. The potential heterogeneity of adolescent NPO, however, has thus far been neglected.

Negative problem orientation is often assessed *via* a well-known self-reported measure called the Negative Problem Orientation Questionnaire (NPOQ) (Robichaud and Dugas, 2005a,b). Although the NPOQ was initially primitively manifested as a uni-dimensional construct related to negative beliefs regarding social problems, it now assesses a comprehensively disruptive cognitive-emotional set of social problems, including perceived threat, self-inefficiency, and negative outcome expectancy (Robichaud and Dugas, 2005a). Xiao et al. (2020) supported a bifactor model of the NPOQ, with a general NPO factor and three domain-specific factors (i.e., perceived threat, self-inefficacy, and negative outcome expectancy). The main purpose of the present study was to explore the latent profiles of adolescent NPO with latent profile analysis (LPA), hypothesizing that potential subgroups of adolescent NPO could be characterized by their NPOQ scores and/or three domain-specific factors.

Latent profile analysis, a person-centered method, can identify latent subpopulations within populations, based on certain variables (Masyn, 2013). Population heterogeneity is explained by the identification of latent profiles that are unique from one another but consist of individuals who are similar with regard to a set of observed variables (Lanza et al., 2013). Theoretically, it is possible that subgroups of adolescents exist as characterized by their different levels of NPO. Recent research has also used person-centered methods to anticipate potential outcomes. For example, Durham et al. (2022) identified three classes of participants with posttraumatic stress disorder who endorsed a posttraumatic sequelae but differed in terms of the level of exposure to trauma (i.e., high, moderate, and low). Similar results were found for eating disorders (Gagliardini et al., 2020), suicidal ideation and behavior (Love and Durtschi, 2021), and intolerance of uncertainty (Boelen and Lenferink, 2018).

Identifying such subgroups would provide valuable information for research and clinical practice. For instance, delineating NPO patterns across potentially emerging subgroups would provide an additional foundation for further clarification of the underlying NPO mechanism and its role in reflecting fundamental anxiety. Moreover, subgroups of adolescents being distinguishable based on NPO profiles underscore the importance of administering tailored interventions to the most problematic groups.

The second purpose of this study was to investigate whether adolescents in different NPO subgroups were distinct in terms of certain cognitive vulnerabilities and psychological symptoms. With regard to cognitive vulnerabilities, worry is examined more as a cognitive vulnerability, such as repetitive thought about negative future events (Borkovec et al., 1998). Thus, this study mainly focused on worry. For psychological symptoms, this research predominately explored stress, anxiety, depressive symptoms, and sleep problems. Given the scarcity of research on latent NPO subgroups, we selected these variables based mainly on previous NPO studies using variable-centered approaches, sufficiently supporting that NPO is a critical cognitive vulnerability for these psychological symptoms (Dugas et al., 2005; Fialko et al., 2012; Hasegawa et al., 2018; Romano et al., 2019). It was thus important to identify the results for the NPO subgroups in terms of these psychological vulnerabilities and symptoms.

Additional significant reasons prompted us to examine associations among different NPO profiles demonstrating these cognitive vulnerabilities and symptoms. First, it would be helpful to enhance our theoretical knowledge of NPO by investigating the distinctiveness of the emerging subgroups in terms of these variables. Specifically, it would be beneficial to understand whether inclusion in different NPO profiles conferred different levels of risk of maladaptive cognitive processes and psychological problems. Second, it would be clinically helpful to explore differences in cognitive vulnerabilities and symptoms among the NPO profiles. In particular, if we could identify NPO profiles as characterized by various cognitive vulnerabilities and symptoms, it would help with determining which NPO aspects or features should be targeted in treatment.

In sum, though NPO has increasingly emerged as an essential construct for understanding deficits in social problem-solving, its heterogeneity has yet to be explored. Through a survey of a large sample of Chinese adolescents (*N* = 2,174) *via* the widely-used NPOQ, this study explored latent subgroups of Chinese adolescents categorized according to NPO indicators, examined the antecedent variables of the NPO profiles, and determined whether they were significantly diverse in terms of particular cognitive vulnerabilities and psychological symptoms. Based on the evidence of latent subgroups found in related research fields (Crow et al., 2012; Boelen and Lenferink, 2018; Xiao et al., 2019), we preliminarily hypothesized that among the different subgroups of Chinese adolescents, there would be at least two subgroups of NPO characterized by relatively low and high NPO on the NPOQ. Given that latent NPO profiles are exploratory, we were hesitant to formulate specific hypotheses related to our second research goal.

## Methods

### Participants

With a stratified cluster sampling (i.e., each class was seen as a cluster, and each grade was considered a level), 2,174 Chinese adolescents were recruited from Fuzhou, Fujian Province in PRC. Approval was obtained from each participant’s head teacher and parents. Invalid questionnaires (characterized by blank or duplicate selections on more than half of the instrument) were eliminated. The remaining 2,050 participants were retained, for a validity rate of 94.3%. Of the valid sample, 1,027 were boys (50.1%) and 1,023 were girls (49.9%); 518 were in the 7th grade (25.3%), 529 in the 8th grade (25.8%), 548 in the 10th grade (26.7%), and 455 in the 11th grade (22.2%). The mean age of the participants was 14.3 years (*SD* = 1.6), with ages ranging from 10 to 19 years. Written informed consent was obtained from participants’ teachers and parents. This study was approved by the Academic Committee of Fujian Normal University.

## Measures

### Negative problem orientation questionnaire

The NPOQ assesses individual negative beliefs regarding social problems, including considering problems as a threat, doubting one’s problem-solving abilities, and being pessimistic about the outcomes (Robichaud and Dugas, 2005a). The NPOQ consists of 12 items, each rated on a five-point Likert scale, with responses ranging from 1 (“not at all true of me”) to 5 (“extremely true of me”). Higher scores indicate a greater NPO. The NPOQ was shown to have excellent internal consistency (*α* = 0.92) and good test–retest reliability (*r* = 0.80; five-week interval) (Xiao et al., 2020). In the current study, the Cronbach’s alpha coefficient for the NPOQ was.93.

### Depression-anxiety-stress scale

The depression-anxiety-stress scale (DASS-21) assesses typical dysphoria and sadness, physiological arousal and fear, and states of tension and stress (Brown et al., 1997). It includes three subscales: depression, anxiety, and stress. Participants rate each item on a four-point Likert scale, with responses ranging from 0 (“did not apply to me at all”) to 3 (“applied to me very much or most of the time”), according to the frequency and severity of symptoms experienced the previous week. Higher scores on the DASS-21 indicate higher levels of negative emotionality. Previous studies have supported the good internal consistency of the DASS-21 and its subscales (Szabo, 2010; Osman et al., 2012). In the current study, the Cronbach’s alpha coefficients for the DASS-21 were 0.94 and for its subscales 0.86, 0.83, and 0.84, respectively.

### Penn state worry questionnaire

The Penn state worry questionnaire (PSWQ) uses 16 items to measure the frequency and intensity of the tendency to worry (Meyer et al., 1990). Participants rate each item on a five-point Likert scale, with responses ranging from 1 (“not typical at all”) to 5 (“very typical”), with 1, 3, 8, and 11 being the reverse scoring items. The total score ranges from 16 to 80, with higher scores indicating a greater tendency to worry after reversing the reverse scoring items. Previous studies have shown that the PSWQ enjoys excellent internal consistency (Meyer et al., 1990). In this study, the Cronbach’s alpha coefficient for the PSWQ was 0.85.

### Pittsburgh sleep quality index

The Pittsburgh sleep quality index (PSQI) is an 18-item self-reported instrument measuring sleep quality according to seven factors: subjective sleep quality, sleep latency, sleep duration, habitual sleep efficiency, sleep disturbance, use of sleeping medications, and daytime dysfunction (Buysse et al., 1989). Each item is rated on a four-point Likert scale, with responses ranging between 0 and 3. Higher scores indicate lower levels of sleep quality. The PSQI shows good internal consistency (Scialpi et al., 2022). In the current study, the Cronbach’s alpha coefficient for the PSQI was 0.63.

### Statistical analysis heterogeneity

The statistical analysis was conducted in Mplus 8.0 and SPSS 25.0. Based on the Mplus 8.0, LPA was used to identify the number of heterogeneity adolescents in NPO subgroups. All items on the NPOQ were subjected to LPA. First, a one-profile model was constructed, followed by two-, three-, four-, and five-profile models. The optimal model was determined by the following indices of model fit: Akaike information criterion (*AIC*), Bayesian information criterion (*BIC*), sample size-adjusted *BIC* (*aBIC*), Lo–Mendell–Rubin (*LMR*), the bootstrapped likelihood ratio test (*BLRT*), and *Entropy* values (Meyer and Morin, 2016). Lower *AIC*, *BIC*, and *aBIC* values indicate a better model fit. *Entropy* values closer to 1 demonstrate a more accurate classification. *LMR* and *BLRT* were adopted to compare the models of *K* profiles to models of *K-1* profiles. The significant *value of p* suggested that the models of *K* profiles had a better fit than the models of *K-1* profiles (Nylund et al., 2007).

After the best profile of the model was selected, latent mixture modeling was further employed to examine the antecedents and consequences of the NPO profiles. The antecedents, which mainly included socio-demographic variables (i.e., gender, age, and grade), were tested using a three-step approach (Asparouhov and Muthen, 2014). The consequence variables, which included worry, depression, stress, anxiety, and sleep quality, were tested by Bolck, Croon, and Hagenaars (BCH) approach (Bolck et al., 2004).

## Results

### Descriptive statistics and correlation analysis

Inter-correlations of the NPOQ, PSWQ, PSQI, and DASS-21 subscales are presented in Table 1. The correlations between the NPOQ and DASS-21 subscales (i.e., depression, anxiety, and stress) were significant (*r_s_* = 0.55 ~ 0.65, *p* < 0.01). The NPOQ correlations with the PSWQ and PSQI were also significant (*r_s_* = 0.49 ~ 0.62, *p* < 0.01; *r_s_* = 0.37 ~ 0.46, *p* < 0.01).

**Table 1:** Inter-correlation and descriptive statistics.

	NPOQ-factor			Depression	Anxiety	Stress	PSQI	PSWQ
	F1	F2	F3					
F1	–							
F2	0.67^**^							
F3	0.71^**^	0.82^**^						
Depression	0.56^**^	0.60^**^	0.62^**^					
Anxiety	0.55^**^	0.57^**^	0.61^**^	0.78^**^				
Stress	0.57^**^	0.61^**^	0.65^**^	0.77^**^	0.83^**^			
PSQI	0.37^**^	0.43^**^	0.46^**^	0.48^**^	0.51^**^	0.52^**^		
PSWQ	0.49^**^	0.59^**^	0.62^**^	0.51^**^	0.58^**^	0.62^**^	0.47^**^	
*Mean*	6.27	9.95	12.57	9.18	11.29	12.81	7.00	50.09
*SD*	3.00	4.51	5.30	9.60	9.46	9.82	3.39	11.55
*CR*	0.76	0.87	0.86	0.85	0.83	0.84	0.89	0.85
*AVE*	0.51	0.62	0.55	0.45	0.41	0.43	0.35	0.32

^*^p < 0.05, ^**^p < 0.01, ^***^p < 0.001. F1, perceived threat; F2, self-inefficacy; F3, negative outcome expectancy; PSQI, Pittsburgh Sleep Quality Index; PSWQ, Penn State Worry Questionnaire; CR, construct reliability; AVE, average variance extracted.

### Latent profile analysis

**Figure 1:**
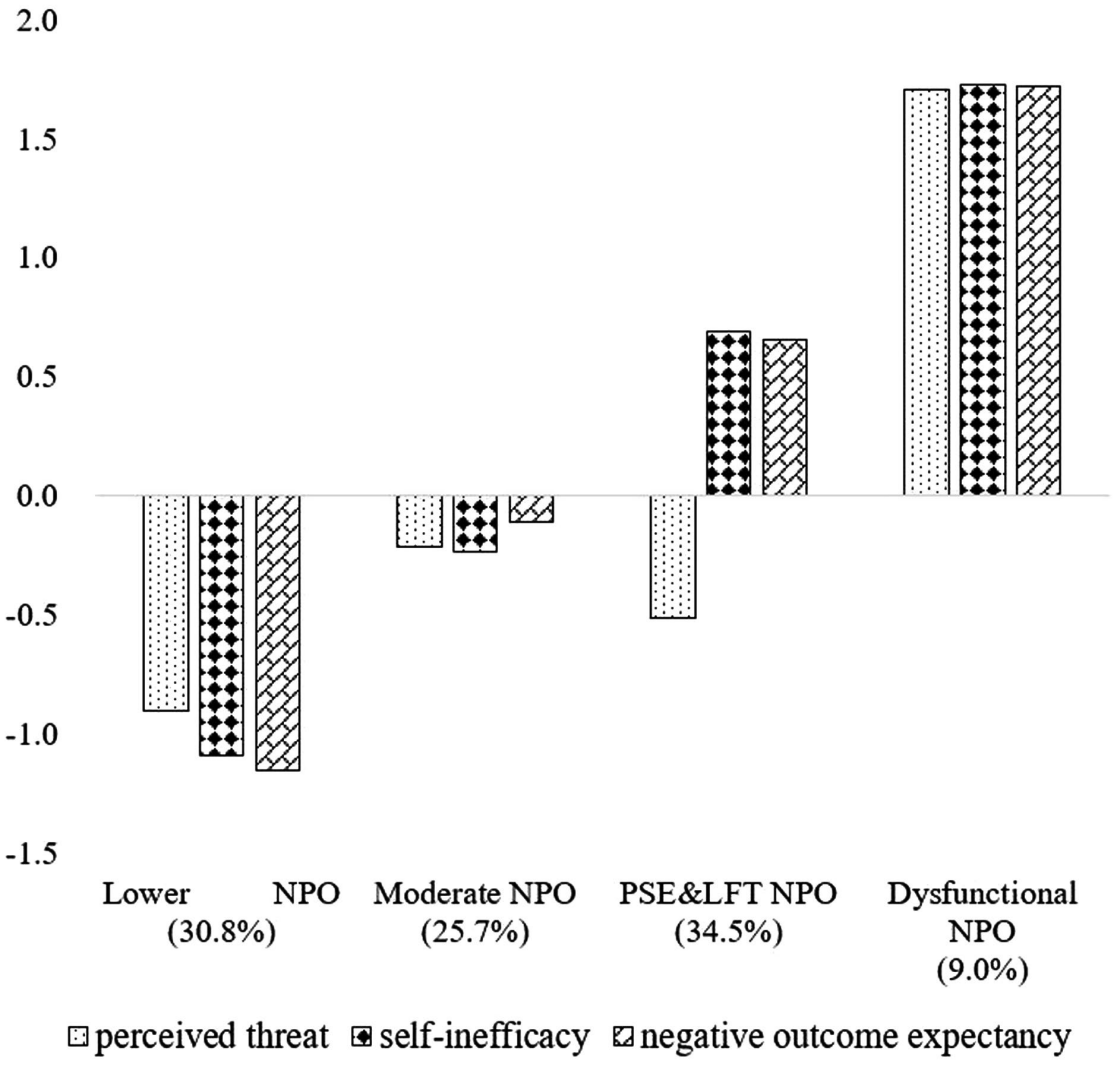
Means of the three domain-specific factors of NPOQ for the four-profile model. profile 1, *lower NPO*; profile 2, *moderate NPO*; profile 3, *SI & NOE*; profile 4, *dysfunctional NPO*; NPO, negative problem orientation; *SI&NOE*, self-inefficacy and negative outcome expectancy.

The fit statistic for the different profile models is presented in Table 2. The results showed that the three-, four-, and five-profile models revealed relatively lower *AIC*, *BIC*, and *aBIC* values as compared to the two-profile model (even with a higher *Entropy*). Meanwhile, the four-profile model showed lower statistical criteria values (i.e., *AIC*, *BIC*, and *aBIC*) as compared to the three-profile model. The statistical criteria for the four-profile model with higher *Entropy* (0.89) and significant *LMR* (*p* < 0.001) and *BLRT* (*p* < 0.001) compared to that of five-profile model. In sum, compared with the three-profile and the five-profile model, the four-profile model indicated demonstrated good discriminability and theoretical interpretability which was accepted. Figure 1 shows the four-profile model for the three domain-specific factors on the NPOQ.

**Table 2:** Fit indices for one-to five-profile models of latent profile analysis.

Profile	*LL*	*FP*	*AIC*	*BIC*	*aBIC*	Entropy	*LMR* (p)	*BLRT* (p)
1	−41299.11	24	82646.22	82781.23	82704.98	–	–	–
2	−36292.54	37	72659.08	72867.23	72749.68	0.93	<0.001	<0.001
3	−34951.31	50	70002.61	70283.89	70125.04	0.91	<0.001	<0.001
4	−34377.74	63	68881.48	69235.89	69035.74	0.89	<0.001	<0.001
5	−34149.32	76	68450.64	68878.19	68636.73	0.88	0.1739	<0.001

*AIC*, Akaike information criterion; *BIC*, Bayesian information criterion; *aBIC*, adjusted Bayesian information criterion; *LMR* (p), value of p of likelihood ratio test index; *BLRT* (p), value of p of bootstrapped likelihood ratio test index; LL, loglikelihood; FP, free parameters.

Profile 1 (30.8%) was characterized most clearly by lowest Z-scores on the perceived threat, self-inefficacy, and negative outcome expectancy, resulting in Profile 1 being named *lower NPO*. Profile 2 (25.7%) was characterized by Z-scores close to but less than zero for the three domain-specific dimensions, leading Profile 2 to be named *moderate NPO*. Profile 3 (34.5%) was the largest and defined primarily by below-average scores for perceived threat and above-average scores for self-inefficacy and negative outcome expectancy. This profile was characterized by obvious self-inefficacy and negative outcome expectancy, and thus was named *SI&NOE*. Profile 4 (9.0%) was the smallest and characterized most notably by significant above-average scores on all dimensions related to NPO. The individuals in the profile tended to be dysfunctional and/or exhibit inhibitive cognitive-emotional patterns in response to social problems; thus, the group was named *dysfunctional NPO*.

### Three-step approach

A three-step approach was taken to analyze the socio-demographic variables (i.e., gender, age, and grade) as possible background characteristics of the NPO profiles. As shown in Table 3, when the *lower NPO* profile was the reference group, the regression coefficients of gender for *SI&NOE* and *dysfunctional NP*O were significantly negative, indicating that a higher proportion of boys were categorized as *SI&NOE* and *dysfunctional NPO* as compared to *lower NPO*. The regression coefficients of grade and age for the *moderate NPO* and *SI&NOE* profiles were also significantly negative, indicating that there were higher percentages of students in the lower grades and younger adolescents in the *moderate NPO* and *SI&NOE* profiles, as compared to *lower NPO*. In addition, when compared to *moderate NPO*, the regression coefficients of gender for *dysfunctional NPO* were significantly negative. They differed significantly for girls and boys, with boys being more likely than girls to be in the *dysfunctional* rather than the *moderate NPO* profile.

**Table 3:** Multinomial logistical regression results predicting the four profiles of NPOQ.

Reference profile		Moderate NPO				*SI&NOE*				Dysfunctional NPO		
		Gender	Grade	Age		Gender	Grade	Age		Gender	Grade	Age
Lower NPO	*B*	−0.08	**−0.15** ^*^	**−0.09** ^*^		**−0.25** ^*^	**−0.11** ^*^	**−0.09** ^*^		**−0.52** ^**^	−0.27	−0.01
	*SE*	0.13	0.06	0.04		0.12	0.05	0.04		0.18	0.08	0.06
	*OR*	0.93	**0.89** ^**^	**0.92** ^*^		**0.78** ^*^	**0.90** ^*^	**0.91** ^**^		**0.60** ^***^	0.97	0.10
Moderate NPO	*B*					−0.17	0.05	−0.01		**−0.44** ^*^	0.13	0.09
	*SE*					0.13	0.06	0.04		0.18	0.09	0.06
	*OR*					0.84	1.05	1.00		**0.64** ^**^	1.13	1.09
*SI&NOE*	*B*									−0.27	0.08	0.09
	*SE*									0.18	0.08	0.06
	*OR*									0.76	1.08	1.09

^*^p < 0.05, ^**^p < 0.01, ^***^p < 0.001. The negative numbers mean there are more boys and more younger adolescents. The bold values means the data is significant.

### BCH approach

The BCH approach was used for the cognitive vulnerability and psychological symptoms variables (i.e., sleep quality, worry, depression, stress, and anxiety), which served as consequence variables for the NPO profiles. As shown in Table 4, worse sleep quality, worry, depression, stress, and anxiety all differed significantly across the four NPO profiles. The mean scores for worse sleep quality, worry, depression, stress, and anxiety were highest for the *dysfunctional NPO* profile; the order of the mean scores was: *lower NPO* < *moderate NPO* < *SI&NOE* < *dysfunctional NPO*.

**Table 4:** Parameter estimates of cognitive vulnerabilities and psychological symptoms on the four profiles, *M*(*SE*).

	*M*(*SE*)	Pairwise comparisons (χ^2^)					
		C1 vs. C2	C1 vs. C3	C1 vs. C4	C2 vs. C3	C2 vs. C4	C3 vs. C4
PSQI	C1 = 4.94 (0.14)	72.50^***^	221.71^***^	145.61^***^	11.56^***^	69.18^***^	46.62^***^
	C2 = 7.24 (0.22)						
	C3 = 8.27 (0.18)						
	C4 = 12.95 (0.65)						
PSWQ	C1 = 38.78(0.57)	55.59^***^	262.13^***^	233.99^***^	34.18^***^	92.12^***^	34.08^***^
	C2 = 46.13 (0.76)						
	C3 = 52.11 (0.60)						
	C4 = 60.71 (1.32)						
Depression	C1 = 2.68 (0.18)	88.83^***^	591.49^***^	552.06^***^	122.95^***^	328.40^***^	133.95^***^
	C2 = 6.55 (0.35)						
	C3 = 12.43 (0.36)						
	C4 = 23.49 (0.87)						
Anxiety	C1 = 4.76 (0.25)	120.65^***^	490.81^***^	607.90^***^	78.57^***^	332.27^***^	161.42^***^
	C2 = 9.50 (0.34)						
	C3 = 14.05 (0.35)						
	C4 = 25.34 (0.80)						
Stress	C1 = 5.62 (0.25)	119.17^***^	600.58^***^	638.76^***^	124.30^***^	345.21^***^	129.14^***^
	C2 = 10.52 (0.35)						
	C3 = 16.43 (0.36)						
	C4 = 26.44 (0.78)						

^*^p < 0.05, ^**^p < 0.01, ^***^p < 0.001. C1, lower NPO, C2, moderate NPO; C3, *SI&NOE*; C4, dysfunctional NPO.

NPO, negative problem orientation; *SI&NOE*, self-inefficacy and negative outcome expectancy.

## Discussion

The main purpose of this study was to explore the heterogeneity of NPO in Chinese adolescents. The results showed that four NPO profiles could be distinguished among the Chinese adolescents (i.e., *lower NPO*, *moderate NPO*, *SI&NOE*, and *dysfunctional NPO*). The three-step regression mixture model showed significant gender, grade, and age differences, and the BCH approach revealed significant differences between the *four profiles* with regard to sleep quality, worry, depression, stress, and anxiety. This study analyzed and discussed the latent profiles, antecedents, and after-effects of NPO, revealing the extent to which they contribute to adolescent NPO.

### Latent profiles of NPO

Four distinct NPO profiles were first distinguished by the three domain-specific factors (i.e., perceived threat, self-inefficacy, and negative outcome expectancy): *lower NPO*, *moderate NPO*, *SI&NOE*, and *dysfunctional NPO*. The *lower NPO* profile included individuals likely to endorse the three domain-specific factors, reflecting a significantly low extent of NPO compared to other individuals, followed incrementally by *moderate NPO*. Importantly, the *SI&NOE* adolescents had not yet viewed problem-solving as threatening; nevertheless, they had a relatively higher probability of endorsing self-inefficacy and negative outcome expectancy. This profile reflects a low level of perceived uncertainty about the future. However, such individuals feature a particular cognitive manifestation of more generalized negative thinking. Furthermore, adolescents with the *dysfunctional NPO* profile were characterized by the highest levels of the three domain-specific factors measured by the NPOQ. They tended to consider problems as threats, question their problem-solving abilities, and be pessimistic about the likely outcomes, reflecting a dysfunctional and/or inhibitive cognitive-emotional pattern in response to social problems.

Compared with the *lower NPO* and *moderate NPO* profiles, the *SI&NOE* and *dysfunctional NPO* profiles explored in this study have more prominent implications. *SI&NOE* individuals have a lower perceived threat to their wellbeing but demonstrate negative cognitive thinking that could also lead to worry and anxiety. This result is consistent with that of social problem-solving, in which worry is associated with one’s confidence and outcome expectancy (D’Zurilla et al., 2004). The *dysfunctional NPO* profile included individuals with an intense response to all three domain-specific factors and strong associations with the symptoms of anxiety and worry (Wilson et al., 2011). Adolescents with this profile are more likely to engage in anxiety and worry, have symptoms related to emotional disorders, and avoid or postpone making an effort to engage in social problem-solving (Becker-Weidman et al., 2010; Anderson et al., 2011). In addition, the *lower* and *moderate NPO* profiles represented relatively reduced NPO and the potential to facilitate proactive problem-solving.

This study also found significant predictive gender and age effects related to the NPO profiles. Boys were more frequently seen in the *SI&NOE* and *dysfunctional NPO* profiles than were girls. This gender difference agrees with prior research showing higher levels of NPO in boys, from which researchers argued that some of the variables could be triggering self-inefficacy and negative outcome expectancy (Barahmand, 2008). Thus, adolescent boys may manifest a more or less upward trend in terms of their negative beliefs about social problems and have more significant doubts regarding whether they can effectively cope. Furthermore, the students in the lower grades and younger adolescents tended to appear in the *moderate NPO* and *SI&NOE* profiles. It can be clearly understood that the reason for this result is due to the result of different psychological development rate of different adolescents. Adolescents may hold up to two polarized beliefs of the social problems they encounter. A part of adolescents tend to believe that problems are solvable, while others doubt their ability to solve problems effectively. In addition, result from their psychological and physiological development are not synchronized, whose transition from immature to mature can be hindered in the period of turbulent development (Hazen et al., 2008).

### Cognitive vulnerabilities and psychological symptoms

This study also investigated the relationships among the latent NPO profiles and certain cognitive vulnerabilities and psychological symptoms. An examination of the differences in cognitive vulnerabilities among the profiles revealed that worry scores were significantly different for all four profiles. The subgroups of NPO manifesting various characteristics of worry can be explained by the cognitive model of worry. Dugas et al. (2005) argued that NPO can be conceptualized as a cognitive process for developing and maintaining worry. Specifically, the *SI&NOE* and *dysfunctional NPO* adolescents tended to have less confidence in their problem-solving abilities, with negative cognitive processes and emotions leading to the avoidance of real problems and engagement in worry, diminishing their social problem-solving abilities and thus leading to negative psychological symptoms. In the cognitive-behavioral therapies (CBT) framework, NPO is a dysfunctional attitude related to intermediate beliefs; from the perspective of problem-solving training (PST), the belief that one cannot solve social problems reflects NPO. Fergus et al. (2015) found that NPO could be a mechanism of change in CBT, in that NPO is viewed as a type of self-efficacy and CBT aims to increase individual self-efficacy. More broadly, according to the person-centered approach, this study further corroborates the cognitive process of NPO.

With regard to psychological symptoms among the latent profiles, we found that compared to participants in the *lower* and *moderate NPO* profiles, the *SI&NOE* and *dysfunctional NPO* adolescents evidenced significantly more severe stress, anxiety, and depressive symptoms and worse sleep quality. Adolescents with *dysfunctional NPO* group have stronger emotional dysregulation and are more prone to repetitive thinking about negative events, which can lead to a variety of psychological symptoms. These findings corroborate prior evidence supporting that NPO can serve as a risk predictor for the development of anxiety, stress, and symptoms of depression, and NPO is an antecedent of a negative affect (e.g., sadness, fear, hostility, and joviality) in adolescents (Ciarrochi et al., 2009; Becker-Weidman et al., 2010; Lee et al., 2018). Moreover, this study found that the *SI&NOE and dysfunctional NPO* adolescents reported worse sleep quality than did the other profiles, and tended to worry excessively about sleep problems. This is consistent with the cognitive model of insomnia, by which cognitive processes promote an individual’s preoccupation with sleep problems (Harvey, 2002; Lin et al., 2017; Tousignant et al., 2019). When individuals perceive the possibility of a bad night’s sleep, they tend to have negative thoughts about sleep problems that influence their individual sleep quality.

## Implications and limitations

In summary, this study is a preliminary attempt to identify subpopulations of individuals characterized by different NPO profiles by using LPA. The heterogeneity of NPO in Chinese adolescents allowed for four NPO profiles to be distinguished according to three domain-specific factors. Adolescents in the *SI&NOE* and *dysfunctional NPO* profiles may suffer from dysfunctional and/or inhibitive cognitive-emotional expressions. The present study contributes to the NPO literature by revealing the important role of social problem orientation, based on health-related correlates (Jaffee and D’zurilla, 2009; Clarke et al., 2016; Suh and Lee, 2018). This study also has several important implications for those seeking to understand adolescents with dysfunctional NPO and other psychological symptoms. For researchers, attention should be focused on the three dimensions of adolescents with dysfunctional NPO to increase positive problem orientation (i.e., perceived challenge, self-efficacy, and positive outcome expectancy). For educators and mental health providers, more tailored interventions should be developed for adolescents in the *dysfunctional NPO* group. Such interventions might include training in problem-solving, cognitive-behavioral therapies, acceptance and commitment therapy, and other effective stress management and prevention methods.

The present study is not without limitations, which may serve as guidance for future research directions. Firstly, no complete set of social-demographic characteristics was tested. For this study, the 7th, 8th, 10th, and 11th grades were included, but the 9th and 12th grades were not considered. In China, students in these two grades are busy preparing for the senior high school entrance examination and the national college entrance examination, respectively. Secondly, only a non-clinical sample of adolescents was considered in this study, which makes it difficult to determine whether the results can be generalized to other populations. Future research should select samples comprised of clinical adolescents. Moreover, a baseline measure to gauge mental health would have provided useful information. Future research should employ a longitudinal design to track the high NPO of the adolescents who participated in this study, further validating the NPO mechanism.

## Data availability statement

The original contributions presented in the study is publicly available. This data can be found at: https://pan.baidu.com/s/1JIm_JizLYczcx47Ob9fDig (accession code: 2ejw).

## Ethics statement

This study was approved by the Academic Committee of Fujian Normal University. Written informed consent to participate in this study was provided by the participants’ legal guardian/next of kin.

## Author contributions

R-ML conceived the original idea for the study. R-ML and X-XX wrote the manuscript. Y-LS and NL revised the manuscript. R-ML and Y-PC supervised this study. All authors contributed to the article and approved the submitted version.

## Conflict of interest

The authors declare that the research was conducted in the absence of any commercial or financial relationships that could be construed as a potential conflict of interest.

## Publisher’s note

All claims expressed in this article are solely those of the authors and do not necessarily represent those of their affiliated organizations, or those of the publisher, the editors and the reviewers. Any product that may be evaluated in this article, or claim that may be made by its manufacturer, is not guaranteed or endorsed by the publisher.
